# Molecular MRI of Atherosclerosis

**DOI:** 10.3390/molecules181114042

**Published:** 2013-11-13

**Authors:** Alkystis Phinikaridou, Marcelo E. Andia, Sara Lacerda, Silvia Lorrio, Marcus R. Makowski, René M. Botnar

**Affiliations:** 1 Division of Imaging Sciences and Biomedical Engineering, King’s College London, 4th Floor, Lambeth Wing, St Thomas’ Hospital, London SE1 7EH, UK; E-Mails: alkystis.1.phinikaridou@kcl.ac.uk (A.P.); mandia@med.puc.cl (M.E.A.); sara.lacerda@kcl.ac.uk (S.L.); silvia.lorrio@kcl.ac.uk (S.L.); marcus.makowski@charite.de (M.R.M.); 2 Radiology Department, School of Medicine, Pontificia Universidad Catolica de Chile, Santiago 8331150, Chile; 3 Department of Radiology, Charite, Berlin 10117, Germany; 4 Wellcome Trust and ESPRC Medical Engineering Center, King’s College London, London SE1 7EH, UK; 5 BHF Centre of Excellence, King’s College London, London SE1 7EH, UK; 6 NIHR Biomedical Research Centre, King’s College London, London SE1 7EH, UK

**Keywords:** MRI, molecular imaging, contrast agent, atherosclerosis

## Abstract

Despite advances in prevention, risk assessment and treatment, coronary artery disease (CAD) remains the leading cause of morbidity and mortality in Western countries. The lion’s share is due to acute coronary syndromes (ACS), which are predominantly triggered by plaque rupture or erosion and subsequent coronary thrombosis. As the majority of vulnerable plaques does not cause a significant stenosis, due to expansive remodeling, and are rather defined by their composition and biological activity, detection of vulnerable plaques with x-ray angiography has shown little success. Non-invasive vulnerable plaque detection by identifying biological features that have been associated with plaque progression, destabilization and rupture may therefore be more appropriate and may allow earlier detection, more aggressive treatment and monitoring of treatment response. MR molecular imaging with target specific molecular probes has shown great promise for the noninvasive *in vivo* visualization of biological processes at the molecular and cellular level in animals and humans. Compared to other imaging modalities; MRI can provide excellent spatial resolution; high soft tissue contrast and has the ability to simultaneously image anatomy; function as well as biological tissue composition and activity.

## 1. Introduction

**Figure 1:**
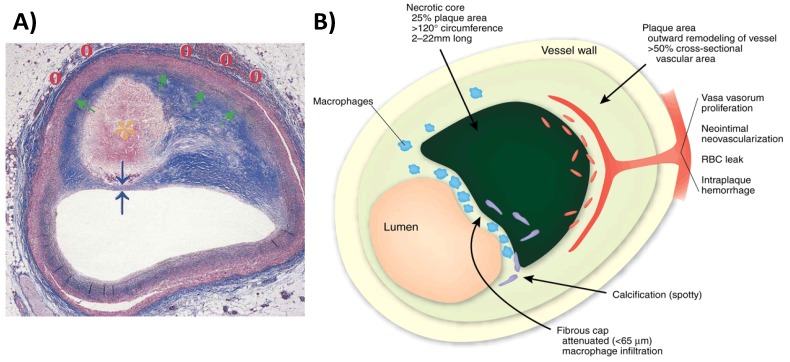
(**A**) Cross section of a rupture-prone coronary artery plaque with a (1) large lipid-rich necrotic core (orange asterisk), (2) thin fibrous cap (blue arrows), (3) expansive remodeling (green arrow), and (4) vasa vasorum and neovascularization (red circles) (adapted from Falk) [4]. (**B**) Schematic of a vulnerable plaque highlighting the features associated with plaque instability (adapted from Narula) [5].

**Figure 2:**
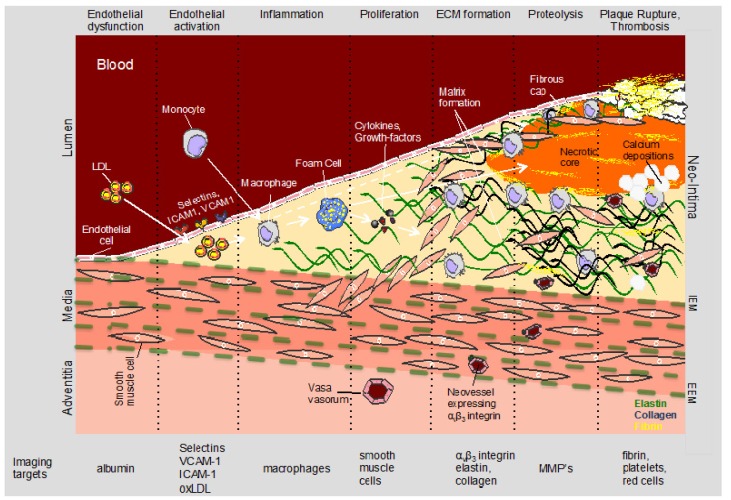
Schematic of biological processes and associated biological targets involved in the initiation, progression and complication of atherosclerosis. MR contrast agents have been shown useful for imaging of highly abundant proteins or cells such as collagen, elastin, fibrin, macrophages or VCAM-1 while PET tracer may be advantageous for visualization of low abundance targets such as MMPs or integrins. MR: magnetic resonance, PET: positron emission tomography, MMP: matrix metalloproteinase.

Despite advances in prevention (e.g., blood pressure control, cholesterol lowering and smoking cessation), risk assessment and treatment, coronary artery disease (CAD) remains the leading cause of morbidity and mortality in Western countries [1]. Fifty percent of men and 64% of women who suddenly die of CVD have no previous symptoms according to the 2008 AHA statistics [2]. The lion’s share is due to acute coronary syndromes (ACS), which are predominantly triggered by plaque rupture or erosion and subsequent coronary thrombosis [3]. Vulnerable plaques are typically large and characterized by a large lipid-rich necrotic core covered by a thin, typically highly inflamed fibrous cap (<65 µm) referred to as thin-cap fibroatheroma (TCFA) (Figure 1). As the majority of vulnerable plaques does not cause a significant stenosis, due to expansive remodeling, and are rather defined by their composition and biological activity (Figure 2), detection of vulnerable plaques with x-ray angiography or nuclear perfusion imaging has shown little success. Non-invasive detection of vulnerable plaques by identifying biological features that have been associated with plaque progression, destabilization and rupture may therefore be more appropriate and may allow earlier detection, more aggressive treatment and monitoring of treatment response.

Intravascular ultrasound (IVUS) and optical coherence tomography (OCT) have emerged as clinical research tools allowing for *in vivo* characterization or “virtual histology” at a resolution of down to 10 μm [6,7]. Both IVUS and OCT are invasive and their application is usually limited to patients referred for coronary angiography. Multi-slice computed tomography allows the detection of calcified lesions and therefore provides a good estimate of the total non-calcified and calcified plaque burden [8] but has low sensitivity for soft plaque characterization and molecular contrast agent detection. Conversely, magnetic resonance imaging (MRI) is a promising non-invasive method for imaging of the coronary artery vessel wall with [9,10] without [11,12] the use of MR contrast agents.

MR molecular imaging with target specific molecular probes has shown great promise for the noninvasive *in vivo* visualization of biological processes at the molecular and cellular level in animals and humans. Compared to other imaging modalities, MRI can provide excellent spatial resolution, high soft tissue contrast and has the ability to simultaneously image anatomy, function as well as biological tissue composition and activity [13].

## 2. Pathophysiology of Atherosclerosis and Molecular Targets

### 2.1. Endothelial Dysfunction

Endothelial dysfunction and damage has long been associated with a greater risk of atherosclerosis and is closely related with a decreased bioavailability of nitric oxide (NO). Decreased production or activity of NO typically leads to impaired vasodilation, increased endothelial permeability and subsequent influx of atherogenic blood proteins, particularly low-density lipoproteins (LDL), which are particularly rich in cholesterol. Although, it has long been known that increased endothelial permeability, with the influx of cholesterol into the intima, is one of the primary events in atherogenesis, non-invasive assessment of endothelial permeability only has recently been demonstrated *in vivo* [14,15,16].

### 2.2. Inflammation

Inflammation is the body’s response to injury or infection. In atherosclerosis, the inflammatory response typically leads to the recruitment and differentiation of monocytes and subsequent digestion and oxidation of LDL by macrophages (Figure 1 and Figure 2). Atherosclerosis primarily affects large and medium size vessels and it is increasingly accepted that atherosclerosis is both a lipid metabolism disorder and a chronic inflammatory [17,18] disease. From autopsy studies it is known that high-risk plaques are characterized by a highly-inflamed, macrophage-rich thin fibrous cap and the presence of a large thrombogenic lipid core (Figure 1) [19,20]. In addition, macrophage infiltration has been associated with stent restenosis [21]. Macrophages (1) secrete inflammatory cytokines that stimulate smooth muscle cell proliferation, migration and subsequent extracellular matrix (ECM) formation; (2) produce proteolytic enzymes that degrade collagen and elastin; (3) and render the growing plaque’s cap thin and susceptible to rupture. Thus macrophages represent an attractive target for molecular imaging at all stages of atherosclerosis and stent restenosis.

### 2.3. Vascular Remodeling

Positive vascular remodeling defined as non-lumen encroaching compensatory enlargement of the vessel wall has been found in the majority of patients dying from myocardial infarction (MI) [5,22] and it has been associated with an excess production of extracellular matrix proteins such as collagen, proteoglycans and elastin. ECM proteins are major components of atherosclerotic lesions [23] accounting for as much as 60% of the neointima and their turnover is a significantly increased in pathologically altered vessel walls [24,25]. ECM formation has also been identified as the principal mechanism of restenosis in various experimental models and in humans after balloon angioplasty or stent placement [26,27]. Hence, the measurement of ECM proteins such as elastin appears to be a promising approach for the detection of subclinical or advanced remodeling in coronary atherosclerosis, stent restenosis and for monitoring treatment response. These findings are also supported by three recent major prospective clinical imaging studies by IVUS and/or coronary CT of patients with CAD that have shown that large plaque burden, small luminal area, the presence of thin-cap fibroatheromas (PROSPECT study) [28], a positive remodeling index (VIVA study) [29], and positive remodeling and low-attenuation [30] were predictors of adverse cardiac events.

### 2.4. Neovascularization and Intraplaque Hemorrhage

The growth of the plaque and the associated increased metabolism, which is due to the infiltration of inflammatory cells, can induce hypoxia and in turn stimulate the proliferation of small neovessels from either the vasa vasorum or the intima or both [31]. Leaky neovessels may facilitate additional infiltration of inflammatory cells. The subsequent release of proteolytic enzymes has been linked to the digestion of the extracellular matrix and weakening of the fibrous cap [32,33]. Moreover, leakiness and rupture of neovessels can lead to intraplaque haemorrhage [34] (Figure 1) or deposition of fibrin [35] and growth of the necrotic core via accumulation of free cholesterol from erythrocyte membranes. Intraplaque hemorrhage and growth of the necrotic core have been identified as strong predictors of plaque vulnerability [3,34]. The role of neovascularisation in plaque destabilization is further corroborated by the finding that the density of vasa vasora is increased in proximal segments of coronary arteries [36] where plaque rupture predominates [37]. Virmani *et al*. [38] have shown that vulnerable (TFCA) and ruptured coronary plaques exhibit a two-fold and four-fold increase in neovascular density, respectively, compared to stable, severely obstructive coronary plaques. Therefore, plaque neo-vascularization seems to be a promising new imaging target for the identification of active and potentially unstable plaque.

### 2.5. Proteolytic Enzymes

Proteolytic enzymes such as metalloproteinases (MMP) play an important role at different stages of atherosclerosis. In the early phases of atherosclerosis, MMPs facilitate the migration of monocytes, macrophages and smooth muscle cells while at the later stages MMPs can cause degradation of extracellular matrix proteins such as elastin and collagen [39,40]. In advanced plaques this can lead to weakening of the fibrous cap thereby promoting plaque rupture and exposure of the thrombogenic core to circulating blood. A major contributor to MMP synthesis are activated macrophages, which are present throughout all stages of plaque development [40]. Other important proteolytic enzymes include the cathepsins S and K, which are potent elastases and involved in the breakdown of elastin, a major structural matrix protein. In advanced atheromatic plaque, cathepsins S and K are highly upregulated and co localize with activated macrophages [39]. Enzyme myeloperoxidase (MPO) is another emerging biomarker and elevated MPO levels have been found in stroke patients [41] and in eroded or ruptured plaques that have caused acute coronary syndromes [42]. MPO is being expressed by granulocyte macrophage colony stimulating (GM-CSF) factor-activated macrophages and was found to produce hypochlorous acid (HOCl), which is a prooxidant and thus increases oxidative stress in plaque [43].

### 2.6. Fibrin

Fibrin represents a well established and clinically relevant molecular target. It has been recognised as an important component of atherosclerotic plaques for more than 100 years with the majority of cells involved in lesion formation displaying procoagulants such as fibrin and/or fibrinolytic factors [44]. It is present in arterial (Figure 2), venous and cardiac thrombi and throughout plaque development where it co-localizes with surface macrophages [35]. Fibrin is also detected in the necrotic core of advanced plaques [35] but it is found at very low concentrations in flowing blood [45]. In addition, fibrin is one of the key elements in thrombus formation following plaque rupture. Therefore, imaging of fibrin has important clinical applications in the diagnosis of different medical conditions including plaque activity, acute coronary syndromes, pulmonary emboli and deep venous thrombosis.

## 3. Molecular Imaging Agents

### 3.1. Probe Detection by MR

Signal intensity in MRI primarily depends on the local values of the longitudinal (1/T_1_) and transverse (1/T_2_) relaxation rate of water protons. Depending upon the pulse sequence, signal usually tends to increase with shorter T_1_ (higher 1/T_1_) and decrease with shorter T_2_ (higher 1/T_2_) relaxation times.

The relaxivities *r*_1_ and *r*_2_, which are commonly expressed in (mM × s)^−1^, indicate the increase in the relaxation rate R_1_ = 1/T_1_ = 1/T_10_ + *r*_1_ * [Gd or Fe] and R_2_ = 1/T_2_ = 1/T_20_ + *r*_2_ * [Gd or Fe] per concentration of contrast agent. [Gd] stands for the local gadolinium while [Fe] for the iron concentration, respectively. The sensitivity to detect a molecular imaging probe depends on the distribution as well as the strength of the probe signal. For probes used in MRI, the strength of the achievable signal is determined by the concentration of the contrast agent at the site it is targeting and the relaxivity of the agent. However, probe signal may not increase proportionately with increases in probe concentration due to competing effects of T_1_ and T_2_/T_2_*. The signal of a steady state gradient echo sequence depends both on T_1_ and T_2_* and can be written as SI = ρ × (1 − E1) × sin(α)/(1 − E1 × cos(α)) × E2 with E1 = exp(−TR/T_1_), E2 = exp(−TE/T_2_*), ρ = proton density, α = flip angle and TR = repetition time. Usually, local concentrations of contrast agents (for example, typically μM to mM for those agents having *r*_1_ relaxivity in the 4–80 mM^−1^s^−1^ range) are needed to alter relaxation rate of water protons sufficiently for detectable signal effects. 

To optimize target-to-background signal ratio distribution of the probe in non-targeted regions or the blood is an important consideration, e.g., it might be necessary to wait for plasma concentrations of the agent to fall sufficiently to distinguish luminal from vessel wall contrast uptake, although imaging methods such as diffusion prepulses may be employed to reduce this requirement [46]. Rapid renal clearance is usually achieved with small molecular weight contrast agents (<25–50 kDa) while nanometer sized particles (10 nm^–1^ μm) are typically cleared by the liver and often exhibit relatively long blood circulation half lifetimes.

### 3.2. T_1_ and T_2_* Mapping

Gadolinium (Gd) based contrast agents usually increase 1/T_1_ and 1/T_2_ in similar amounts (*r*_2_/*r*_1_ ≅ 1–2) [47,48,49] whereas iron particle based contrast agents have a much stronger effect on increasing 1/T_2_ (*r*_2_/*r*_1_ > 10) [50]. Gadolinium based contrast agents therefore lead to a positive contrast effect (detected as an increase in signal intensity or brightness) whereas iron particle based contrast agents usually cause a negative contrast effect (detected as a decrease in signal intensity or darkness). MR pulse sequences that emphasize differences in T_1_ and T_2_ are commonly referred to as T_1_ and T_2_ weighted sequences [51,52]. Apart from their effect in increasing 1/T_2_, iron particles also increase 1/T_2_* due their effect on the local magnetic field B_0_ thus causing local field inhomogeneities ΔB_0_.

**Figure 3:**
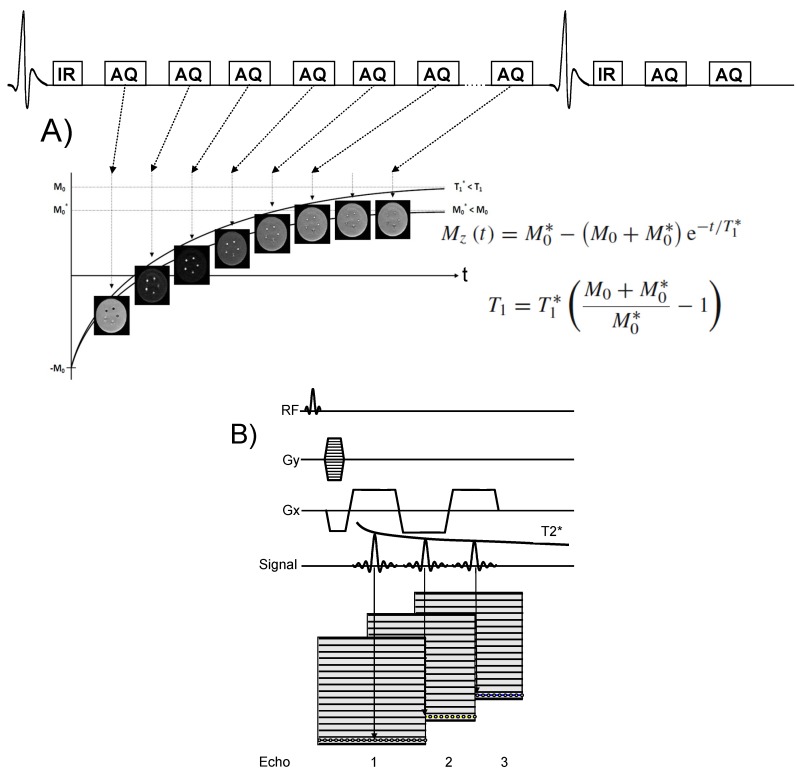
(**A**) Schematic of inversion recovery Look Locker (LL) T_1_ mapping sequence. The inversion pulse precedes the imaging sequence (AQ), which allows pixel wise sampling of the recovery of the Mz magnetization of e.g., tissues, fluids or blood. A least square fit of the measured Mz values to the Bloch equation allows pixel wise estimation of T_1_ and estimation of the local contrast agent concentration [Gd^3+^]. A T_1_ correction is usually performed as the repetitive use of RF pulses alters the recovery of the M_z_ magnetization leading to a lower value for the steady state magnetization, M_0_* < M_0_ and thus to a shorter value for the relaxation time T_1_* < T_1_. (**B**) Schematic of T_2_* mapping sequence using Cartesian k-space sampling. Acquisition of multiple echoes allows sampling the T_2_* decay envelope and thus to estimate the T_2_^*^ relaxation time of tissues and blood.

This additional effect leads to even more severe signal decay. T_1_ and T_2_* mapping MRI sequences allow quantification of T_1_ and T_2_* thereby providing the local contrast agent concentration in the examined tissue of interest. T_1_ mapping is often performed with a Look Locker sequence (Figure 3A) where a non-selective inversion pulse is followed by a train of image acquisitions in order to sample the recovery of the Mz longitudinal magnetization (Mz(t) = Mss − (M_0_ + Mss) × exp(−t/T_1_*), Mss = steady state longitudinal magnetization, M_0_ = equilibrium longitudinal magnetization, Mz(t) = longitudinal magnetization at time t, T_1_* = apparent T_1_). T_2_ mapping is usually performed with a multi echo gradient echo technique (Figure 3B). 

### 3.3. T_1_ Contrast Agents

Most MR contrast agents are based on either gadolinium (Gd) complexes [48,49] or iron oxide particles [50]. Gd(III) is ideally suited for use as an MRI contrast agent because it not only has seven unpaired electrons but also the symmetry of its electronic states produces an electron spin relaxation time slow enough to interact significantly with neighboring water protons [49].

**Figure 4:**
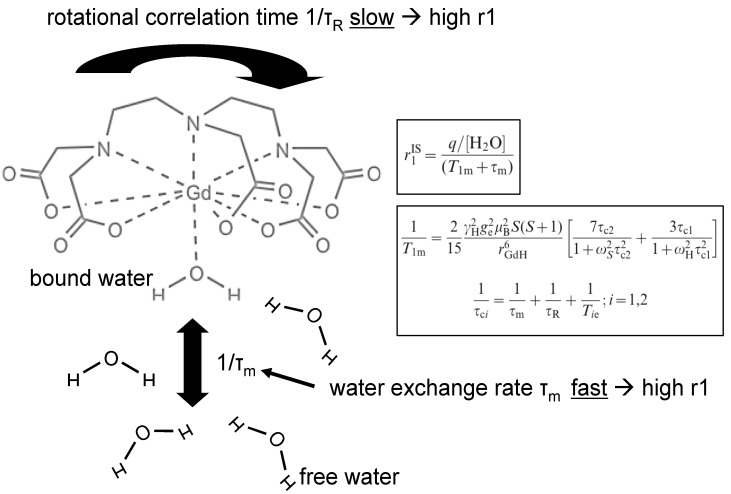
Schematic of water exchange between bound and free water of a gadolinium chelate. The proton bound to gadolinium exchanges its magnetic properties with the free water leading to a shortening of T_1_ in the vicinity of the contrast agent. Fast water exchange leads to a more efficient energy transfer between Gadolinium and free water, thereby creating a higher relaxivity *r*_1_. The amount of T_1_ shortening is given by the relaxivity (*r*_1_) of the contrast agent, which depends on the number of bound protons, the water exchange rate (1/τ_m_) and the T_1_ relaxation time of bound water (T_1m_) [49]. T_1m_ itself depends on several factors including the distance to the gadolinium atom and the rotational correlation time (1/τ_r_). Upon binding to a large protein or cell, 1/τ_r_ significantly decreases thereby increasing *r*_1_ and thus increasing the T_1_ shortening effect of the contrast agent. Construction of macromolecular agents (e.g., dendrimers) is another means of slowing down the rotational motion of Gd-compounds. The relationship between the relaxation time of bound water T_1m_ (black box) and the inner sphere relaxivity r1^IS^ (black box) is given by the Solomon Bloembergen equations [56] which are described in more detail in [49,57].

**Figure 5:**
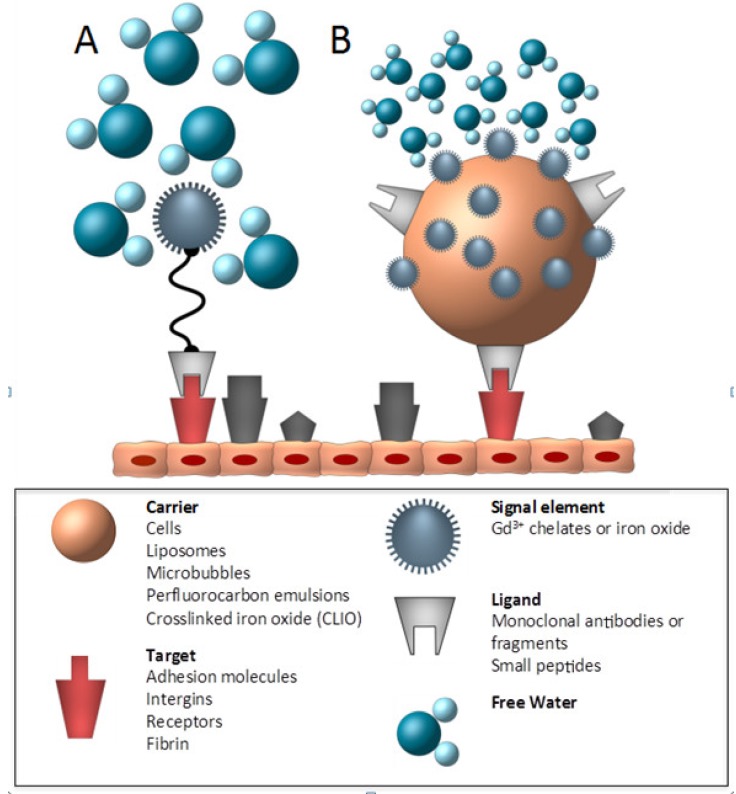
Schematic of a small molecular weight targeted contrast agent and a targeted nanoparticle. (adapted from Choudhury) [62]. The basic components of a target specific contrast agent consist of a ligand that binds to a specific target and a signal element, which, in case of MR, is made of a Gd^3+^ chelate or an iron oxide. These two basic components can be directly (**A**) linked to each other or may be attached to or incorporated within a larger nanoparticle (carrier) with the advantage of a higher payload of paramagnetic or supramagnetic agents as shown in (**B**).

Relaxivity of target specific contrast agents is determined primarily by the rotational correlation time (τ_R_) (Figure 4). To enhance relaxivity (~0.1 ns for approved agents), a variety of efforts in contrast agent design have focused on increasing this parameter. τ_R_ is lengthened by formation of conjugates between the metal ion complex and slowly moving structures such as proteins, polymers or dendrimers. Molecular MRI probes frequently involve attachment of Gd complexes to small ligands (e.g., small molecules, peptides) that in turn attach to large slowly moving targets (e.g., proteins); thus, lengthening of the rotational correlation time is accomplished, providing a convenient means of amplifying the detection of contrast agents positioned at molecular targets (Figure 5). Since the unbound fraction of the molecular probe will maintain a lower *r*_1_, a high target-to-background signal ratio can be achieved for small molecular weight contrast agents with renal clearance. This has been termed receptor-induced magnetization enhancement (RIME) [53,54]. An early example of this type of contrast agent is gadofosveset, an intravascular contrast agent (Lantheus Medical Imaging, North Billerica, MA). Gadofosveset reversibly binds to albumin in plasma. When bound to albumin the relaxivity increases to 42 mM^−1^s^−1^ from the 6.6 mM^−1^s^−1^ observed in phosphate buffered saline (PBS) at 20 MHz [49].

To further amplify the MRI signal for the detection of very low abundant targets, the number of Gd per ligand and/or carrier system can be increased [55]. This effect can be exploited for peptide and antibody probes as well as for nanoparticles (Figure 5). However, the magnitude of the effect, and thereby the increased signal level, is substantially higher for nanoparticles (e.g., ~4 Gd/peptide *vs.* ~20 Gd/antibody *vs.* ~300,000 Gd/300 nm nanoparticle). Rigid binding between the Gd molecules has been found essential to obtain high relaxivity Gd-ligand assemblies. This principle applies only to a small fraction of the Gd atoms in these complexes, which are located at the surface of the particle and therefore able to interact with surrounding water molecules. A drawback of the antibody (55–150 kDa) and nanoparticle (10 to 500 nm) based probes is their large size potentially limiting accessibility to target sites and preventing rapid blood clearance.

### 3.4. T_2_/T_2_* Contrast Agents

The synthesis and application of stable, nanosized iron oxide particles for use as MR contrast agent have been extensively studied [50,58]. Depending on their size, iron oxide particles have different effects on 1/T_1_ and 1/T_2_. Superparamagnetic iron oxide (SPIO) particles produce much stronger effect in 1/T_2_ than in 1/T_1_ so they are best displayed with T_2_-weighted scans [58]. SPIO particles produce a marked disturbance in surrounding magnetic field homogeneity, especially apparent when an inhomogeneous distribution produces a T_2_* susceptibility effect.

On the other hand, USPIOs (ultra-small superparamagnetic particles of iron oxide) or VSOPs (very small iron oxide particles) have a stronger effect on 1/T_1_, so they can be used for T_1_-weighted imaging of the great vessels (e.g., aorta, carotids) or the coronary arteries [59,60,61]. The T_1_ effect is typically more readily distinguishable from potential artifacts produced by tissue interfaces, hemorrhage or signal cancellations at water-fat interfaces, which all produce negative contrast effects. At higher concentrations or when accumulated in the tissue of interested, USPIO’s and VSOP’s exhibit a strong T_2_* effect leading to a focal signal void.

### 3.5. Mechanism of Targeting

A specific receptor, molecule or cell type can be targeted by a contrast agent by either passive or active means. Contrast agents can either bind selectively to molecular targets (“active” targeting, e.g., receptor binding) or favor particular tissues or cell types due to specific distribution characteristics (*i.e.* “passive” targeting).

#### 3.5.1. Passive Targeting

Examples of passive targeting agents are iron oxide particles (like SPIOs [63], USPIOs [64], Monocrystalline Iron Oxide Nanoparticle (MIONs) [50] or VSOP’s [65]) that are taken up by components of the reticuloendothelial system (RES) and phagocytosed by plaque macrophages as seen in apoE mice, hyperlipidemic rabbits [66] and humans [67].

#### 3.5.2. Active Targeting

A particular molecule, receptor or cell type can be identified by targeting specific molecular markers. Potential targets may be selected from a variety of molecular targets associated with various diseases like cell surface markers such as αvβ3 or VCAM-1 expressed on endothelial cells or the scavenger receptor on activated macrophages as well as molecular markers (e.g., proteins, collagen, elastin, fibrin, enzymes) that are specifically expressed in certain stages of disease [68]. However, even if contrast enhancement with a targeted probe is observed, results must be interpreted with caution since non-targeted contrast agents may also accumulate in areas of interest due to, e.g., unspecific binding [46,69,70,71]. Therefore, careful use of control and competition experiments are necessary to help establish the basis of contrast effect.

## 4. Molecular Imaging Applications

### 4.1. Endothelial Dysfunction and Activation

**Figure 6:**
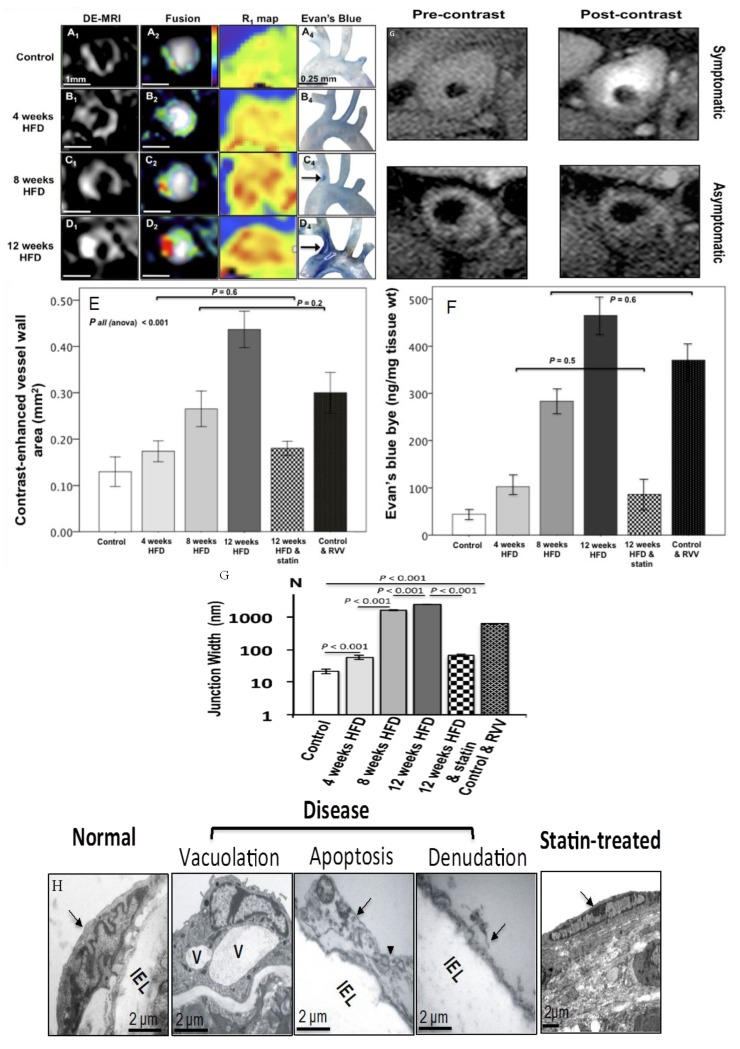
Late gadolinium enhancement (LGE) MRI and T_1_ mapping of endothelial permeability throughout the course of plaque development in high fat diet (HFD) fed apoE−/− mice using an albumin binding contrast agent, gadofosveset, (**A_1_**–**D_1_** and **A_3_**–**D_3_**). Evan’s Blue dye staining of the artic arch and brachiocephalic artery shows good agreement (**A_4_**–**D_4_**). Quantification of contrast enhancement on LGE MRI (**E**) was also in good agreement with Evan’s Blue dye quantification (**F**). Gap junction width measured by transmission electron microscopy (TEM) showed a significant increase after the commencement of HFD and normalization in animals treated with statins (**G**) [16]. TEM of endothelial cell (EC) morphology (**H**) showed gradual disease development starting with vacuolation and almost complete EC denudation after 12 weeks of HFD and normalization in mice treated with statins. Clinical data (G) demonstrate increased gadofosveset uptake in patients with symptomatic carotid artery disease, which correlated with increased neovessel density and macrophage count (adapted from Phinikaridou) [16].

In the early stages of atherosclerosis, in response to abnormal flow conditions or systemic inflammation, endothelial permeability is often increased and function impaired. Endothelial dysfunction is closely associated with a decreased bioavailability of nitric oxide (NO), and results in impaired vasodilation, increased endothelial permeability and subsequent influx of atherogenic blood proteins, particularly LDL. Due to the importance of the endothelium in maintaining vascular homeostasis, it represents an important imaging biomarker for the early detection of atherosclerosis. Recent studies by our group have demonstrated the ability of an albumin binding contrast agent, gadofosveset, to non-invasively monitor changes in endothelial permeability of the brachiocephalic and carotid artery in HFD fed apoE−/− mice *in vivo* (Figure 6) [16]*.* Lobbes *et al.* observed increased gadofosveset in patients with symptomatic carotid artery disease, which was correlated with the density of leaky neovessels (Figure 6) [72]. Hays *et al.* demonstrated the ability of MRI to measure endothelial function in the coronary arteries non-invasively after isometric handgrip exercise and found a correlation between impaired vasodilation and increased plaque burden [14,15]. 

Endothelial dysfunction also leads to the expression of adhesion molecules on the endothelial surface. These proteins support the adhesion and extravasation of pro-inflammatory cells out of the blood and into the inflamed tissue. Therefore, these proteins display interesting targets for molecular imaging [18].

**Figure 7:**
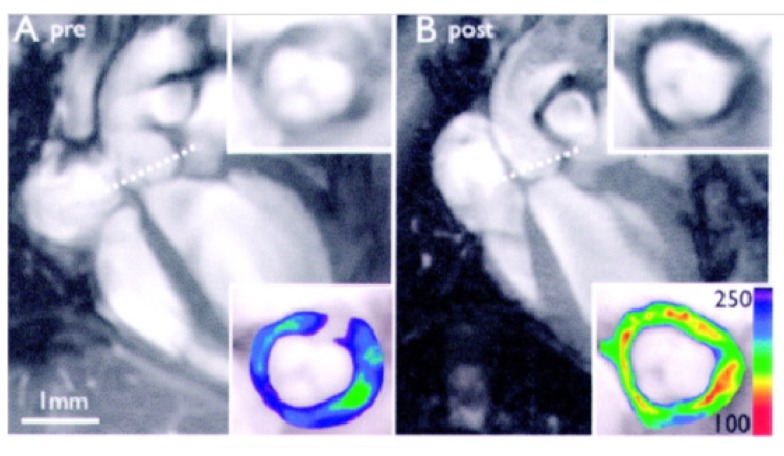
*In vivo* imaging of atherosclerotic inflammation using a peptide targeting VCAM-1 (VINP-28) performed in the aortic root of apoE−/− mice prior to (**A**) and after (**B**) administration of VINP-28. Considerable decrease in signal intensity is seen on the transverse MR images of the aortic root wall (grayscale insets). The contrast-to-noise ratio is significantly increased after the administration of the probe (color insets), demonstrating noninvasive imaging of VCAM-1–expression on endothelial cells and macrophages of atherosclerotic vessel segments (adapted from Nahrendorf) [76].

VCAM-1 has been at the center of attention and several generations of MRI probes were developed to image this target. In the latest generation a probe derived from phage display and coupled to an iron oxide was developed. This led to successful imaging of VCAM-1 in the aortic root of atherosclerotic mice (Figure 7) [73,74]. However, targeting of other adhesion molecules may also be achieved with alternative agents based on proteins, peptides or small molecules specific for e.g. selectins or other integrins conjugated to MRI contrast agents [75].

### 4.2. Macrophages

**Figure 8:**
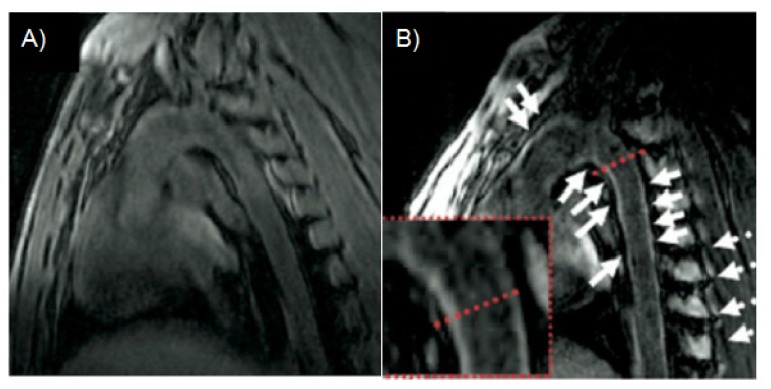
Imaging of macrophage burden using an off-resonance imaging sequence (IRON, inversion recovery with ON-resonant water suppression). (**A**) Before the injection of MION tissue is homogeneously suppressed using the IRON sequence. (**B**) 48 h after the injection of MION, intraplaque iron oxide particles generate areas of positive contrast on IRON images, which were in good agreement with areas of iron deposition on corresponding histological slices. The magnitude of positive signal enhancement correlated with the number of macrophages determined by histology. MION: Magnetic iron oxide nanoparticle (adapted from Korosoglou) [80].

Macrophages can be imaged with MRI by several methods. The most common approach is based on the use of ultra-small superparamagnetic iron oxide particles (USPIO) stabilized with either steric (e.g., dextran) or electrostatic (e.g., citrate) coating and has been demonstrated both in experimental animal models and in patients [67]. Those nanoparticles are typically phagocytosed by plaque resident or blood born macrophages but the exact mechanism leading to the accumulation of iron oxide particles in atherosclerotic plaque remains unknown. The first successful demonstration of non-invasive macrophage imaging was by Ruehm *et al.* using a rabbit model of atherosclerosis. Electron microscopy (EM) confirmed the uptake of iron particles by plaque resident macrophages [66]. More recently, macrophage imaging was performed in apoE−/− and wild-type mice using antibody labeled paramagnetic micelles targeted against the macrophage scavenger receptor. MR signal was increased in the plaque of apoE−/− mice was in good agreement with the density of macrophage [77,78]. Clinical studies in symptomatic patients scheduled for carotid endarterectomy showed preferential USPIO uptake in macrophage-rich plaques as confirmed by histology and EM [67]. Furthermore, USPIO uptake was higher (75%) in ruptured and rupture-prone lesions compared to stable lesions (7%). The development of positive contrast techniques such as inversion recovery on resonance (IRON) also allows visualization of USPIO’s as hotspots, which facilitates detection of USPIO containing macrophages (Figure 8) [79].

### 4.3. Extracellular Matrix

**Figure 9:**
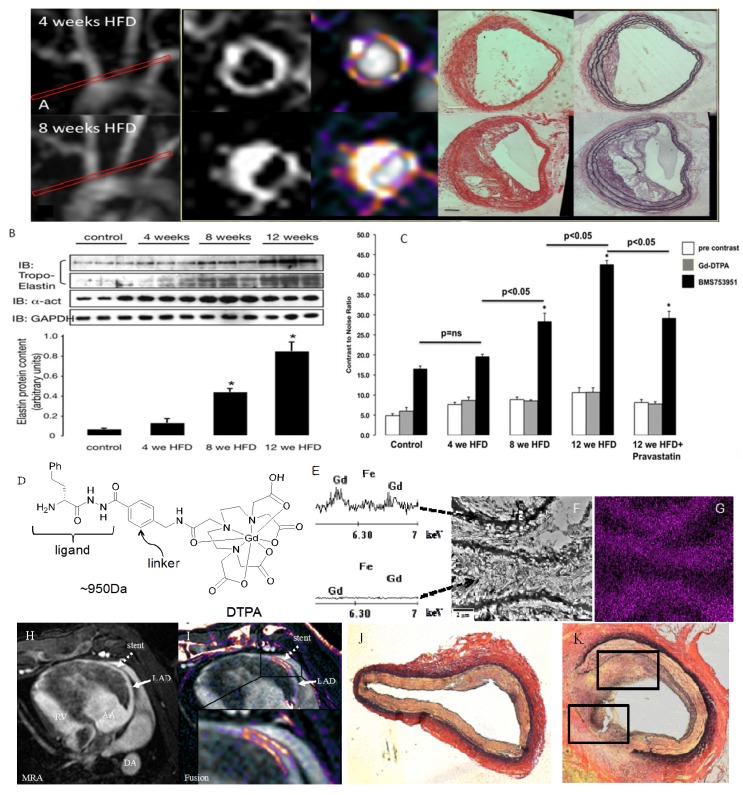
(**A**) MRI of extracellular matrix remodeling in an apoE−/− mouse model of accelerated atherosclerosis at 4 and 8 weeks after commencement of HFD using an elastin specific MR contrast agent (**D**), ESMA (Lantheus Medical Systems, North Billerica, MA, USA). (**B**,**C**) Contrast-to-noise values after ESMA injection increased with the duration of the HFD, which was paralleled by an increase in tropoelastin by western blotting. Electon microscopy of elastin fibers (**F**) and X-ray spectroscopy of gadolinium (Gd) (**E**,**G**) showed preferential uptake of ESMA along the elastic fibers with little to no uptake in-between elastin fibers (F,G). (F) ESMA MRI of mechanical coronary wall injury after MR lucent stent placement (**H**) showed focal signal enhancement in the stented area (**I**), which was in agreement with extracellular matrix remodeling on histology (**J**,**K**) (adapted from Makowski [80] and von Bary [81]).

ECM proteins are the major components of atherosclerotic lesions [23] accounting for as much as 60% of the neointima and their turnover is increased in pathologically altered vessel walls [24,25]. With the development of a small molecular weight elastin specific gadolinium based MR contrast agent (ESMA), MRI of remodeling at all stages of atherosclerosis has become feasible and has been recently demonstrated in an apoE−/− mouse model of accelerated atherosclerosis (Figure 9) [80]. Successful translation into the coronary arteries has then subsequently been demonstrated in a swine model of coronary injury [81] and may provide a new means of non-invasive assessment of coronary artery wall remodeling and plaque burden in patients with suspected coronary artery disease or in-stent restenosis. Other matrix proteins that have been explored for imaging of atherosclerotic plaque include collagen. Promising results for plaque and aortic aneurysm detection were reported in three recent studies [82,83,84].

### 4.4. Intraplaque Hemorrhage and Thrombus

The intrinsic T_1_ and T_2_ differences of diseased tissues may allow for non-contrast enhanced imaging of both thrombus [85,86,87], pulmonary embolism [88] and vulnerable plaque [89,90,91]. In the case of thrombus, this technique is based upon the presence of methaemoglobin, which is formed from haemoglobin by oxidative denaturation to the ferric (Fe^3+^) during the acute phase of thrombus development. Methaemoglobin has paramagnetic properties, which results in shortening of the T_1_ relaxation time [85,86,92]. *In vitro* studies have demonstrated that there is a linear relationship between the concentration of methaemoglobin and T_1_ shortening [93]. On T_1_-weighted images, a sufficient T_1_ shortening effect allows generation of positive contrast between a thrombus or, e.g., pulmonary embolus [88] (bright) and surrounding tissues (dark). Previous studies have used this T_1_-weighted imaging technique to detect thrombi [94,95,96,97,98,99,100] however, this effect declines over time as the thrombus becomes organized [101].

### 4.5. Neovascularization

In more advanced atherosclerotic lesions new blood vessels start developing, preferentially in the adventitia [58,102,103]. The presence of these newly formed vessels has been associated with plaque inflammation and instability. Their activated endothelial cells express unique surface proteins (e.g., αvβ3), which are not expressed by resting endothelial cells of blood vessels in non-diseased tissues [104].

**Figure 10:**
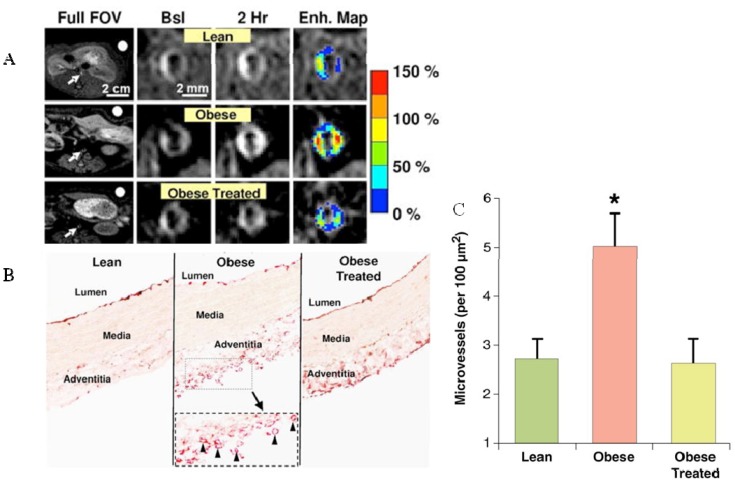
(**A**) *In vivo* MRI of neovascularization 2 hours after injection of avb3-binding paramagnetic nanoparticles in lean, obese and obese treated mice. Contrast enhancement was increased in obese mice and was reduced to normal levels (of lean mice) after treatment with the appetite suppressant, benfluorex. (**B**) Staining of von Willebrand factor shows high density of neovessels in the adventitia of obese mice. (**C**) Quantification of adventitial microvessel density demonstrates a more than two fold increase in obese mice and a normalization after benfluorex treatment (adapted from Winter) [106].

Two strategies have been demonstrated to visualize neovascularization. One approach relies on targeting one of the specific surface markers. This has been done with gadolinium-containing liposomes targeting the αvβ3 integrin, which successfully detected the local increase in angiogenesis in a rabbit model of atherosclerosis [105]. More recently the same group could demonstrate that neovascularization is increased in plaques of obese rats and normalizes in obese rats fed the appetite suppressant benfluorex (Figure 10) [106]. Alternatively, it is possible to directly measure the effect of increased blood flow in the adventitia due to these newly formed blood vessels by applying dynamic contrast-enhanced (DCE) MRI methods [107].

### 4.6. Proteolytic Enzymes

Several groups have investigated the feasibility of direct imaging of proteolytic enzymes during plaque development and in other diseases. Recent work by Lancelot *et al.* and Hyafil *et al.* demonstrated successful imaging of MMPs using an MMP inhibitor conjugated to a gadolinium chelate (P947) both in mouse and in rabbit models of experimental atherosclerosis [108,109]. Binding of P947 was stronger to excised MMP-rich compared to MMP-poor human carotid plaque. A good affinity for purified MMPs was also observed. Ronald *et al.* demonstrated the feasibility of imaging myeloperoxidase (MPO) in the aortic wall of New Zealand White rabbits fed a high cholesterol diet using bis-5-hydroxytryptamide diethylenetriamine pentaacetate gadolinium (MPO(Gd)) contrast agent. Focal areas of increased image intensity on MPO(Gd) MRI colocalized with myeloperoxidase-rich areas infiltrated by macrophages [110].

### 4.7. Fibrin

**Figure 11:**
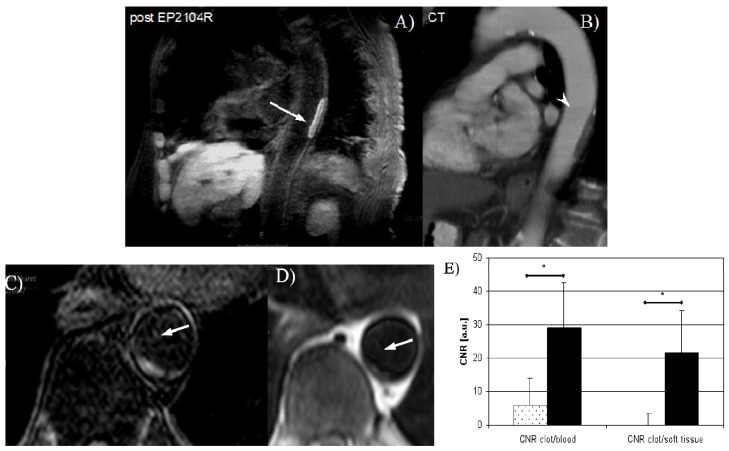
Human thrombus imaging using EP-2104R, a fibrin binding contrast agent, in a patient with aortic thrombus. A mural aortic thrombus is well delineated on inplane post EP-2104R black blood MR images (**A**) while differentiation between thrombus and vessel wall is poor on multi slice CT images (**B**). Excellent aortic thrombus visualization on cross-sectional delayed enhancement images post EP-2104R (**C**) while differentiation between thrombus and vessel wall is poor on pre contrast black blood turbo spin echo (**D**) or gradient echo (**E**) MR images [117].

In several studies, gadolinium labeled fibrin-avid nanoparticles [111] and small peptides (EP-2104R) have been successfully used for imaging of thrombus in the jugular vein [112], aorta [113], the pulmonary [114] and coronary arteries [114,115]. More recently, intraplaque fibrin imaging has been demonstrated in a mouse model of accelerated atherosclerosis [116]. Gadolinium concentrations as low as ~50 μM (*r*_1_ ≅ 21 mM^−1^s^−1^ per Gd) were sufficient for the ready visualization of mural and lumen encroaching thrombus as well as intra plaque fibrin. This is due to the higher relaxivity of EP-2104R compared to non-targeted conventional contrast agents like Gd-DTPA (*r*_1_ ≅ 3–5 mM^−1^s^−1^ per Gd). The administered dose was 4–7.5 μmol/kg, much lower than that for conventional non-targeted Gadolinium based contrast agents (typically ~0.1 mmol/kg). This agent has also been used successfully to image thrombosis in patients (Figure 11) [117].

### 4.8. Lipids

Another approach to image atherosclerotic plaque is to take advantage of its high lipid content by using highly lipophilic contrast agents. An example of a lipophilic, macrocyclic, chelate of gadolinium is Gadofluorine M (Bayer Schering Pharma AG, Berlin, Germany). This contrast agent has a longer circulation half-life than conventional chelates and is more lipophilic. In a rabbit model of atherosclerosis, a high accumulation in plaques was shown [46]. Recent publications have also shown co-localization of the contrast agent with neovessel-rich regions, another feature of vulnerable plaque [118]. A further example of lipid targeted contrast agents includes gadolinium containing high-density lipoprotein (HDL)-like nanoparticles. Those HDL-like nanoparticles (7–12 nm diameter) directly and specifically home to atherosclerotic plaques, as they are responsible for the reverse cholesterol transport from peripheral tissues. Moreover, unlike other nanoparticles, HDL like nanoparticles do not trigger immunoreactions and are not recognized by the reticuloendothelial system (RES) making them an ideal imaging agent [119]. To directly target macrophages, recombinant reconstituted high-density lipoprotein HDL particles (rHDL) have been conjugated with a carboxyfluoresceine-labeled apolipoprotein E-derived lipopeptide, P2fA2 [120]. rHDL- P2fA2 particles were shown to accumulate in lipid and macrophage rich regions of plaque in apoE−/− mice and being taken up more avidly by plaques with higher macrophage density than non-targeted rHDL particles [120].

## 5. Limitations

A challenge for clinical translation of target specific MR contrast agents is the low sensitivity of MRI (μM) compared to PET (nM) and thus the relatively high local concentration (~50 μM) needed for signal detection. Due to this limitation, only imaging of high abundance biological targets is feasible with MR small molecular weight contrast agents. One means of increasing the payload is the use of nanoparticles, and several groups have demonstrated successful use of gadolinium labeled or iron oxide based nanoparticles for imaging of e.g., αvβ3 [121], VCAM-1 [74] or macrophages [66]. The typically injected clinical dose is in the range of 0.1 mmol/kg but can be even lower for target specific contrast agents due to the increased relaxivity upon binding. To address the recent concerns about the safety of gadolinium, newer MR contrast agents use DOTA instead of DTPA chelates due to the significantly better stability of DOTA chelates. In spite of these limitations, a few target specific MR contrast agents have been approved for clinical use (e.g., gadofosveset (albumin binding) and ferumoxytol (iron oxide nanoparticles)) and several promising new agents are in the pipeline (e.g., elastin and fibrin binding contrast agents).

## 6. Conclusions

MR molecular imaging of various biological processes in atherosclerosis has been validated in small and large animal models and has also been successfully demonstrated in pilot patient studies. Targeted imaging probes consisted of small molecular weight, mostly Gd-peptide compounds, as well as nanosized particles including micelles, liposomes, perfluorocarbons and iron oxide nanoparticles. Small molecular weight contrast agents were mostly used to image high abundance targets (fibrin, elastin, collagen and albumin) while nanosized particles showed potential for imaging of low abundance and endothelial targets or cells such as E-selectin, αvβ3, VCAM-1 or macrophages. With the approval of gadofosveset, a blood albumin binding agent, the recent approval of ferumoxytol, a superparamagentic ultra small iron oxide particle, and promising phase II results of EP-2104R, a fibrin binding contrast agent, molecular MRI has entered the clinical arena. With ongoing contrast agent and MR sequence developments, there is great hope that molecular MRI can improve diagnosis of atherosclerosis and monitor therapy response.
